# The temporal response of a glioma cell population to irradiation: modelling the effect of dose and cell density

**DOI:** 10.1098/rsos.241917

**Published:** 2025-04-30

**Authors:** Marianne Billoir, Delphine Crepin, Stéphane Plaszczynski, Basile Grammaticos, Olivier Seksek, Mathilde Badoual

**Affiliations:** ^1^ CNRS/IN2P3, IJCLab, Université Paris-Saclay, Orsay, France; ^2^ IJCLab, Université Paris Cité, Orsay Île-de-France, France

**Keywords:** irradiation, mathematical modelling, cancer, time-lapse microscopy

## Abstract

Time-lapse fluorescence microscopy experiments were performed to track the cell density of F98 glioma cells under varying radiation doses and initial cell densities. Based on these results, a compartmental model characterizing the temporal response of a cancerous cell population to single-dose radiation therapy was developed. This model reproduces very well all the experimental data, with only three free parameters (and four others that are fixed). It allows us to have access and follow the evolution of different cell populations after irradiation, in particular, the senescent and repaired cell populations. From these different cell populations, surviving fractions could also be estimated. Most importantly, our model allows us to analyze and quantify an inhibition effect (or cohort effect) of the dead and senescent cell populations on the regrowth of the repaired one.

## 1. Introduction

Gliomas constitute the majority of malignant brain tumours in adults. Their ability to diffusely infiltrate neighbouring brain tissue makes them very difficult to treat and they are typically associated with a poor prognosis [1]. Radiation therapy (RT) stands out as the most commonly administered oncological treatment with over 50% of cancer patients receiving it at some stage during their therapy [2]. To mitigate radiation-induced toxicity in normal tissues, RT is typically fractionated into smaller doses of 1.8 to 2 Gy per daily session over 5 to 7 weeks [3]. This approach—developed on an empirical basis [4] —allows surrounding non-cancerous tissues to recover between treatment fractions. Nevertheless, despite remarkable advancements in delivering radiation to tumours and the wide exploration of various dose escalation and fractionation schemes, there has been no definitive improvement in the long-term survival of glioma patients [5]. The diffusive nature of glioma plays a key role in their poor prognosis. Tumour cells migrate into normal tissues around the tumour, making it difficult to identify the margins of the tumour. A better knowledge and modelling of the effect of radiation on cell populations, with different cell densities and different doses, could help improve the therapeutic results of radiotherapy for gliomas.

The most widely-used model that allows to estimate the cell survival under a given dose is the linear quadratic model (LQ model) [6,7]. Its main advantage is that it has only two parameters [8]. However, even though it has undergone thorough experimental and clinical validation over the years [9–11], these parameters still lack a clear biological meaning [11,12]. Moreover, their estimation is conducted from experiments performed in very specific conditions (in particular with a very low cell density) called clonogenic assays [13]. In [14], it was shown that cell cooperation could impact the plating efficiency— i.e. the percentage of viable cells that successfully grow and form colonies after being plated onto the bottom of the plate—and led to significantly undervalued errors in these assays. *In vivo*, the situation is even more complex: the response to irradiation of cells in tumour can be modulated by a large number of factors, such as the tumour’s genetic and spatial heterogeneity (structure of tumours into proliferative areas at the border and hypoxic ones at the centre), the microenvironment, intercellular communication [15].

Another limitation of the LQ model is its inaccuracy for high doses (typically above 10 Gy) [16]. If this was usually not a problem due to the low doses employed therapeutically, new therapies are now proposing the use of higher doses such as the FLASH [17], minibeam (MBRT) [18] and microbeam (MRT) [19] therapies. The latter shows real promise in improving treatment efficacy while sparing normal tissues. Moreover, a study of the effect of high doses [20] also revealed that cell survival decreases with cell density. Since in tumours, the cell density can be very high, it is of great interest to perform experiments at high doses and high cell densities.

But, the main drawback of the LQ model lies in the fact that it is not a dynamic model. It gives the fraction of surviving cells at only one fixed time-point, usually chosen around two weeks after irradiation. Yet, *in vivo*, the response of tumours to radiation is not static but, on the contrary, involves time scales spanning from minutes (for short-time repair) to weeks (late apoptosis, long-time tissue response). For example, in [21] it is shown that low-grade glioma’s radius in patients can decrease for months and even years after RT before a systematic recurrence. In order to be able to understand this very long tumour and tissue response, it is crucial to model the time-dependent response of cell populations.

Thus, in order to better adapt the treatment choice of delivery, schedule and doses, we need a better characterization of the tumour’s response to radiation than the one offered by the conventional models used in radiobiology. A strong effort of the community has emerged in the past few years to develop dynamic models of the cellular response to RT. They typically contain a large number of parameters and do not resolve the issue at high doses [22–27]. In [28,29] the authors proposed a simple mechanism-based mathematical model describing the time-resolved response of tumour cells to both single-dose and fractionated radiation. Their model involves an ad hoc function for late cell death. At another scale, while numerous models describing the spatial structure of the tumour exist, cell death resulting from RT is often either phenomenologically treated or estimated using the LQ model [30,31]. We believe that models of cell death should stay simple enough to be incorporated into more complex models of tumour growth.

In this work, we propose a simple, biologically motivated, compartment-based mathematical model characterizing the dynamical response of a cancerous cell population to single-dose RT. To validate our model, we carry out *in vitro* time-resolved fluorescence microscopy experiments to retrieve the cell density of F98 glioma cells over time after exposure to a single dose of radiation, spanning a large range of doses ([0−15] Gy) and seeding concentrations ([1.1 $10^{4}$ - 3.52 $10^{5}$] cells/well with 6-well plates, i.e. [0.55 $10^{4}$ - 1.76 $10^{5}$] cells/ml). We show that it is possible to get an excellent agreement between the model and the data with only three free parameters for every radiation dose and seeding density. We also highlight the effect of the initial cell density on the regrowth dynamic.

## 2. Material and methods

### 2.1. Experiments

#### 2.1.1. Cell culture

The F98 cell line is a glial-like cell isolated from the brain of a rat with an undifferentiated malignant glioma that can be used as a rat brain tumour model [32]. F98 glioma cells are obtained from the American Type Culture Collection (ATCC CRL−2397, Manassas, USA) and cultured with Dulbecco’s Modified Eagle Medium (DMEM, Gibco™, Thermo Fischer Scientific, France) containing L-glutamine, supplemented with 10% fetal bovine serum, 1% Pyruvate, 1% Peniccilin and 1% HEPES. The culture media is also supplied with 1% G418 (Gibco™, Thermo Fischer Scientific, France), a specific antibiotic to keep only successfully transfected cells.

The cells are grown in culture flasks (ClearLine) and maintained at 37°C in a humidified 5% CO${,}_{2}$, 95% air atmosphere in a BINDER incubator. Confluent cultures are washed once with Dulbecco’s Phosphate Bufferred Saline and incubated 3 min at 37°C with 0.05 % trypsin-EDTA (Gibco™, Thermo Fischer Scientific, France) to obtain cell suspensions. The mean passage number of cells used in the experiments is 20±5, the fluorescence tending to disappear beyond 30 passages. The day before irradiation (20±2 h), the cells are seeded on 6-well plates (SPL Life Sciences, Deutscher, France) from 1.1 $10^{4}$ to 3.52 $10^{5}$ cells per well with 2 ml of medium per well. In control conditions, the most concentrated wells reach the carrying capacity before one week (around 3 days for the highest seeding concentration).

#### 2.1.2. Transfection

The transfection protocol is described in electronic supplementary material, §2. After this step, the nuclei of the cells are fluorescent.

#### 2.1.3. Radiation treatment and imaging

After seeding, the cells are grown overnight to allow for attachment and recovery to the growth phase before irradiation. To deliver various doses of radiation, we use the Xrad 320 Dx irradiator of the RadeXp platform (Institut Curie, Orsay, France). The cells are irradiated at either 0, 5, 10 or 15 Gy at 1.1 Gy/min. Right after treatment, 1 ml of medium is added in each well so that nutrients are not a limiting factor of the growth during the time-lapse of the experiment.

About 3 h after treatment, phase-contrast images and red fluorescent images are acquired with the Incucyte S3 live imaging system (CEA, Fontenay-aux-Roses, France) using a 10 x objective with an exposure time of 250 ms at the 550 nm excitation wavelength for the fluorescence. Nine pictures per well are taken every 90 min up to ≃144 h post-irradiation, covering a total area of ≃2.18 mm${,}^{2}$ per well.

#### 2.1.4. Clonogenic assay

The classical LQ model describes the surviving fraction of cells S after exposure to a radiation dose D as:


(2.1) $$\begin{array}{lcr} & S=e^{-\alpha D-\beta D^{2}} & \end{array}$$

where α [Gy${,}^{-1}$] and β [Gy${,}^{-2}$] characterize the cell-specific sensitivity to the dose.

We conventionally characterized the F98 cell line by performing a clonogenic assay based on the protocol given in [13] and we calculated α and β from the measurements of S at 9 days for various doses (see electronic supplementary material, §1 for more details).

### 2.2. Numerical methods

#### 2.2.1. Image analysis

To determine the number of nuclei in each microscopy image, we first compute the background of each picture at initial time with the Python library for Source Extraction and Photometry [33,34]. The latter is subtracted from the image, which is then divided by the root mean square intensity to get the signal-to-noise ratio. A Gaussian filter is applied to smooth and reduce noise followed by a Laplacian filter (LoG) to identify nucleus edges. Both steps are performed using the multidimensional image processing tools of the Scipy Python library [35]. By imposing a threshold, we retrieve the inside of the edges, which we then label using the connected-component labelling method of the OpenCV-Python library [36]. To reject noise, any connected component with less than 10 pixels is discarded. Finally, local maxima in each connected component are identified using the dilation and erosion analysis methods available in the OpenCV-Python library. This means that we must calibrate two parameters: the value of the smoothing σ and the value of the threshold applied s. We find that the values σ=3.5 and s=0.0375 give the best compromise for every dose with a mean relative error of 6.3±0.7% (estimated from the manual count of cells in a given field, see electronic supplementary material, §3).

#### 2.2.2. Numerical implementation of the models and fitting procedure

The ODE model was implemented in Python using the Adams/BDF method with automatic stiffness detection and switching [37,38] of the Scipy Python library [35] for the resolution. For each cell response curve, we determine the set of parameters that best fits our data by numerically performing a multidimensional minimization of the objective function:


(2.2) $$\chi_{\nu }^{2}(\theta )=\frac{1}{\left(n-k\right)}\sum_{i=1}^{n}{\left[\frac{C_{\text{data}}(t_{i})-C_{\text{mod}}(t_{i},\theta )}{\sigma_{\text{data}}(t_{i})}\right]}^{2}$$

where n is the number of data points, k is the number of free parameters, $C_{\text{data}}(t_{i})$ denotes the measured cell density at time $t_{i}$, $\sigma_{\text{data}}(t_{i})$ represents the standard deviation of the mean cell density measured at time $t_{i}$, $C_{\text{mod}}$ is the model-given value of the cell density and θ represents the set of model parameters. This step is carried out with the optimization package of the SciPy Python Library [35].

For each parameter, $10^{4}$ initial random values were used. From those different fits corresponding to different seeds we selected the fit showing the smallest mean squared error. This procedure was repeated several times, to check that the optimization function always converges towards the same minimum. Doing so, we ensure the robustness of the fit, by increasing the probability that the optimum that is found is a global minimum, rather than a local one.

## 3. Results

### 3.1. Image analysis and cell response curves

**Figure 1.**
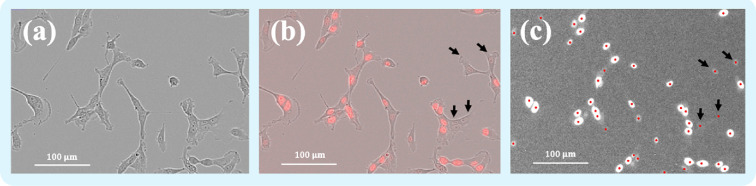
Zoom of microscopy images (10 x objective via Incucyte) of F98 cells in control conditions at time *t* = 0. Here, $C_{0}=3.95$ $10^{-4}$ cells/µm${,}^{2}$. Panel (a) shows the phase-contrast image. Panel (b) shows the corresponding composite image of the red fluorescence microscopy image with the phase contrast image. Finally, panel (c) shows the result of the detection algorithm on the fluorescence image displayed in grey levels with pixels in the range of the mean intensity value ±2 times the standard deviation. Each red point corresponds to the position of a local maximum detected by the algorithm. The black arrows on panels (b) and (c) show nuclei with a low level of fluorescence that are correctly detected.

An example of the nuclei detected by the algorithm described in 2.2.1 is shown in figure 1 with a zoomed red fluorescence microscopy image at 0 Gy and its corresponding phase-contrast (to see the whole cells) and composite images. Some of the nuclei display a low level of fluorescence—for example the cells pointed out by the black arrows in figure 1 on panels (b) and (c)—yet the algorithm is able to detect them.

We plot the time evolution of the cell density (which we assume to be the same than the nuclei density) for each dose and each initial cell density. In every figure of the article, the cell density at each data point is averaged over three independent experiments and the error bars represent the standard deviation. The error due to detection is two to three times smaller (6 % compared to 15–20% depending on doses and times) so we did not include it in the error bars.

**Figure 2.**
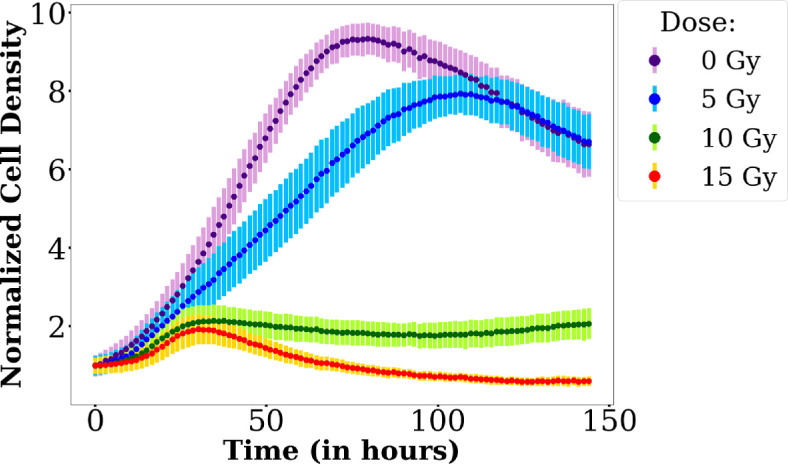
Time evolution of cell density normalized by the initial cell density for each radiation dose starting from the same seeding concentration (the highest one among the 6 wells, 3.52 $10^{5}$ cells/well on 6-well plates).

Instances of cell response curves normalized by the initial cell density for the different doses are presented in figure 2. At cell density around $1.310^{-3}$ cells/µm${,}^{2}$ (or four times the initial cell density of the most concentrated well), around 50% of the confluence at 0 Gy is reached. After that point, other sources of cell death (such as lack of space) can compete with cell death due to irradiation: the cell density even decreases at large times, for 0 and 5 Gy. To avoid the need to account for cell death due to environmental factors, we decide, in what follows, to focus on the evolution of the cell population for cell densities larger than this value. We choose the threshold value $C_{T}=1.3\;10^{-3}$ cells/µm${,}^{2}$.

**Figure 3.**
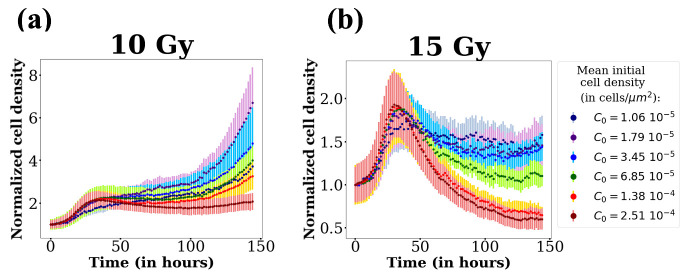
Normalized cell densities versus time for 10 Gy (a) and 15 Gy (b), for every seeding concentration. Each curve represents a different seeding concentration starting from 1.1 $10^{4}$ cells/well up to 3.52 $10^{5}$ cells/well on 6-well plates, each concentration being the double of the previous one. The resulting initial cell densities are presented on the left part of the figure.

The cell response curves for 10 Gy and 15 Gy normalized by the initial cell density are also displayed in figure 3 (respectively panels (a) and (b)) for all initial cell densities.

In figure 2, all the curves display a similar shape: an initial increase, a maximum and a decrease. However, for the 0 Gy and 5 Gy cases, the maximum of the curves occurs after 60 h (at confluence) whereas at 10 Gy and 15 Gy, the curves have their maximum at the same time value around 35 h and well before confluence. This local maximum is reached at the same time for all initial densities (see figure 3). At 10 Gy, and even at 15 Gy for several initial densities, a regrowth is noticed after several days.

### 3.2. Model of cell growth with and without irradiation

Exposure to radiation causes DNA damage to the cells, prompting the activation of the DNA damage response. This, in turn, triggers the induction of cell-cycle checkpoints and initiates DNA repair mechanisms [39]. While undergoing repair, certain processes—such as transcription and cell-cycle progression—are temporarily halted to prioritize and concentrate on the repair tasks [40–43], especially for DNA double-strand breaks (DSBs), the main repair of which typically occurs in the first 24 h after RT. After a transient delay, most cells resume proliferation [44] at either their normal rate (if they have been properly repaired) or at a slower rate due to residual damages inducing the activation of checkpoints [40,42]. Thus, the repair process takes time and particularly, it sometimes leads to misrepairs—the actual main driver of radiation-induced cell death [23]. Usually, cells with remaining or misrepaired DNA damage undergo one or several cell divisions before being driven to an irreversible non-proliferative fate. This process—arising from altered mitoses and irreparable chromosome damage [45]—is called mitotic catastrophe [39,41,44,46]. It either leads to cell death or a state of irreversible growth-arrest with metabolic activity referred to as senescence [46,47].

The biological processes that take place in cells after irradiation are complex and involve very different scales (from molecules to cell population, for a review, see [48]). In our aim of modelling the fate of the cell population, we focus here on the cellular scale and the different possible states of the cells after irradiation, as described in [48]: damaged cells in a transient cycle arrest, cells that undergo mitotic catastrophe, then die or become senescent or cells that manage to repair and resume proliferation. We divide the response of the population to radiation in two phases. The first one, the ‘repairing phase’, right after irradiation, is a phase where some of the cells are still repairing the main damages induced by the treatment (principally DSBs), while others already resumed their cycle. The proliferation of the population, being almost nil at the beginning of the experiment, increases slowly. We model the evolution of the cell density C during this phase by a proliferation coefficient $\frac{1}{C}\;\frac{dC}{dt}$ increasing linearly with time:


(3.1) $$\frac{dC}{dt}=(\frac{k_{d}}{T_{a}})tC$$

where $T_{a}$ denotes the duration of the repairing phase and $k_{d}$ the final growth rate of the population.

**Figure 4.**
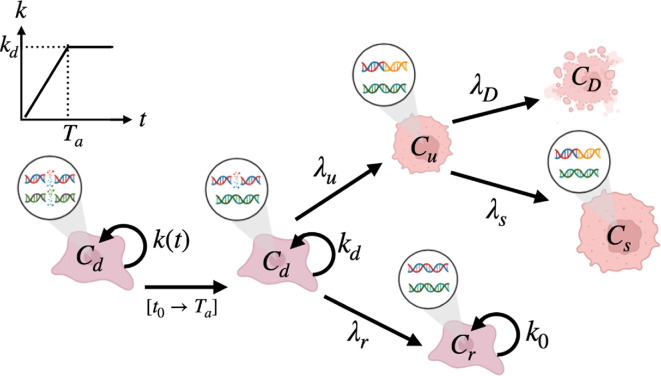
Illustration of the compartments of the model. Above each of them are represented two double strands of DNA to exemplify their respective cells' state. On the left is schematized the first phase of the response with a growth coefficient k that increases linearly from $t_{0}$ to $T_{a}$ until it reaches the value of $k_{d}$. During this repairing phase, cells repair most of the DSBs, which is illustrated by one of the two DSBs represented repaired in the compartment $C_{d}$ at $t>T_{a}$. The second DSB is still unrepaired to signify that not all damages are repaired yet. In the compartment of ‘repaired cells’ $C_{r}$, all damages are well repaired whereas, in the compartment of ‘unrepaired cells’ $C_{u}$, the damages are misrepaired as illustrated by the orange part of the double strands of DNA. Finally, the compartment $C_{D}$ represents the cells which are dead because of irradiation and $C_{s}$ is the compartment of senescent cells that survive despite the misrepair but loose their ability to proliferate. The figure was created with BioRender.com.

For the second phase, the ‘regrow-or-die phase’, we model the evolution of the cell density through compartments illustrating the different fates of the cells (see figure 4). We consider five compartments: the initial compartment $C_{d}$ where cells are still considered to be in a ‘damaged’ state but can proliferate with a proliferation coefficient $k_{d}$ (only the main damages have been repaired at this stage), a compartment of well-repaired cells $C_{r}$, a compartment of unrepaired cells $C_{u}$, a compartment of senescent cells $C_{s}$ and finally, a compartment of cells dead after irradiation, $C_{D}$. From the initial compartment, cells can either become ‘well-repaired’ and go to the compartment $C_{r}$ at a rate $\lambda_{r}$ or become ‘badly-repaired’ and go to the compartment $C_{u}$ at a rate $\lambda_{u}$. Once in the compartment $C_{r}$, cells resume a normal growth rate $k_{0}$ whereas in the compartment $C_{u}$, they either die at a rate $\lambda_{D}$ or become senescent at a rate $\lambda_{s}$ (mitotic catastrophe).

Therefore, the evolution equations of the different compartments are:


(3.2) $$\begin{array}{rl} \frac{dC_{d}}{dt} & =-\gamma C_{d}\; \end{array}$$

(3.3) $$\begin{array}{rl} \;\frac{dC_{r}}{dt} & =k_{0}C_{r}+\lambda_{r}C_{d} \end{array}$$

(3.4) $$\begin{array}{rl} \;\frac{dC_{u}}{dt} & =\lambda_{u}C_{d}-\delta C_{u} \end{array}$$

(3.5) $$\begin{array}{rl} \frac{dC_{s}}{dt} & =\lambda_{s}C_{u} \end{array}\;$$

(3.6) $$\begin{array}{rl} \frac{dC_{D}}{dt} & =\lambda_{D}C_{u} \end{array}\;$$

where $\gamma =\left(\lambda_{u}+\lambda_{r}-k_{d}\right)$ and $\delta =\lambda_{D}+\lambda_{s}$.

In the literature, cell proliferation is usually modelled with a logistic term, which accounts for the increasing lack of resources (e.g. lack of space) [49]. For the sake of consistency, we added this logistic term in all the proliferation processes, i.e the proliferation of the $C_{d}$ and the $C_{r}$ cells. The previous equations (3.1)−(3.3) become:

for $t<=T_{a}$:


(3.7) $$\frac{dC}{dt}=(\frac{k_{d}}{T_{a}})tC(1-\frac{C}{C_{\text{max}}})$$

and for $t>T_{a}$:


(3.8) $$\begin{array}{rl} \frac{dC_{d}}{dt} & =-(\lambda_{u}+\lambda_{r})C_{d}+k_{d}C_{d}(1-\frac{C}{C_{\text{max}}}) \end{array}\;$$

(3.9) $$\begin{array}{rl} \frac{dC_{r}}{dt} & =k_{0}C_{r}(1-\frac{C}{C_{\text{max}}})+\lambda_{r}C_{d}. \end{array}\;$$

Equations (3.4)–(3.6) do not change and complete the system of equations that we have to solve.

In experiments, dead cells detach and disappear from the field of view. Since we can only count the cells that remain attached to the bottom of the plate and our objective is to compare the experimental cell density with a simulated one, we do not include the dead cells in the total computed cell density: $C=C_{d}+C_{r}+C_{u}+C_{s}$. The differential equation for C is then:


(3.10) $$\frac{dC}{dt}=(k_{0}C_{r}+k_{d}C_{d})(1-\frac{C}{C_{\text{max}}})-\lambda_{D}C_{u}.$$

We can check theoretically that the model with irradiation converges towards a usual model of cell growth in absence of radiation—i.e. with an exponential growth and a logistic term—when the dose tends to zero. Starting from the model with irradiation, if the dose is set to zero:

— the repairing phase disappears, so $T_{a}=0$
— no cells die nor become senescent due to radiations, so $\lambda_{u}=0$. With the initial conditions $C_{u}(t=0)=0$, $C_{s}(t=0)=0$, we find that $C_{u}(t)=0$, $C_{s}(t)=0$ and the total cell density reduces to : $C(t)=C_{d}(t)+C_{r}(t)$
— no change is observed in the growth rate of the population, so $k_{d}$ is simply the normal growth coefficient denoted as $k_{0}$.

With this, we find $dC/dt=k_{0}(C_{d}+C_{r})(1-C/C_{\text{max}})=k_{0}C(1-C/C_{\text{max}})$, which is the non-irradiated growth. Here, there are still ‘damaged cells’ in the expression of C. However, one has to remember that the damages to the cells decrease with the dose. Therefore, at 0 Gy, the damages are equal to zero when the dose is zero, and thus the $C_{d}$ population is identical to the $C_{r}$ population.

### 3.3. Model parameters and fitting results

In this section, we describe how we can reduce the number of parameters by fixing some of them and we show the final fitting results.

#### 3.3.1. Measuring and fixing parameters: $T_{a}$ and γ

**Figure 5.**
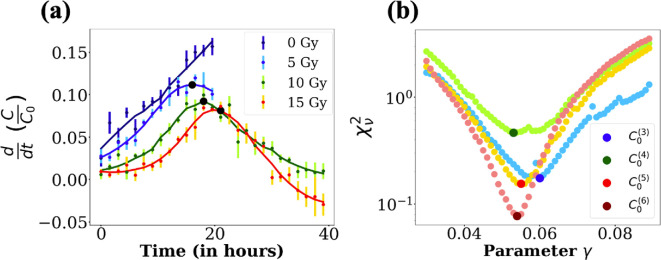
Panel (a): Derivative of the normalized cell density averaged over the six initial cell densities for each dose as a function of the time. In plain lines are represented the smoothed curves using the Savitzky−Golay filter implemented in the Scipy Python library. The black points indicate the maximum found for each curve. The corresponding values of time to each maximum (which coincides with $T_{a}$) are the following: for 0 Gy, 0 h; for 5 Gy, 16 h: for 10 Gy, 18 h; for 15 Gy, 21 h. Panel (b):$\chi_{\nu }^{2}$ values as a function of γ for the four highest initial cell densities in the 15 Gy case. For each value of γ taken in [0;0.2]h${,}^{-1}$ with a step of $10^{-3}$ h${,}^{-1}$, the model is fitted to the 15 Gy curve using 100 random draws for the initial parameters' values. The values of γ obtained for each minimum and for each initial cell density are the following: $C_{0}^{\left(3\right)}:6.00$ $10^{-2}$ h${,}^{-1}$; $C_{0}^{\left(4\right)}:5.30$ $10^{-2}$ h${,}^{-1}$; $C_{0}^{\left(5\right)}:5.50$ $10^{-2}$ h${,}^{-1}$; $C_{0}^{\left(6\right)}:5.40$ $10^{-2}$ h${,}^{-1}$. The initial densities are identified by the number they have been given in the parameter values electronic supplementary material, table in §6 (from the lowest to the highest).

The first measurable parameter is the duration of the repairing phase $T_{a}$. Indeed, our experimental curves of the cell density versus time display an inflexion point around t≃20 h, that corresponds to a maximum in the derivative of the experimental curves (see figure 5). Now, in the model, the first inflexion point of C(t) corresponds to the transition between the repairing phase and the regrow-or-die phase (at time $t=T_{a}$). More particularly, the time point $t=T_{a}$ corresponds to a maximum in the derivative of the function C(t) (see electronic supplementary material, §4 for a more detailed justification of this point). Hence, the position of the maximum of the derivative of the experimental cell density can be used to estimate $T_{a}$, the duration of the repairing phase. We find that for 0 Gy $T_{a}=0$h, for 5 Gy $T_{a}=16$h, for 10 Gy $T_{a}=18$h and for 15 Gy $T_{a}=21$h (see figure 5). As expected, we verify that $T_{a}$ tends to zero with the dose.

We now focus on the parameters γ and δ. Since we cannot monitor the density of dead cells $C_{D}$ (because they detach from the bottom of the plate), we cannot find the value of $\lambda_{D}$ by fitting the data. Therefore, instead of having two different characteristic times 1/γ and 1/δ, with δ being unknown, we prefer to simplify the model and fix γ=δ. Setting γ=δ is not a strong constraint, it only fixes the value of $\lambda_{D}$ to $\gamma -\lambda_{s}$.

With γ=δ, we can rewrite the equation (3.4) as:

(3.11) $$\frac{dC_{u}}{dt}=\lambda_{u}C_{d}-\gamma C_{u}.$$

For high doses (15 Gy), the cell densities are low and we can neglect the logistic term in the proliferation. Equations (3.2)–(3.6) can easily be solved (see electronic supplementary material, §4). The total cell density C(t) reaches its maximum almost at the same time as the function $C_{u}(t)$, i.e. at time $T_{\text{max}}=T_{a}+1/\gamma$. Therefore, from the measure of the time of the maximum, we can obtain estimation of γ. In experimental data, for the 10 and 15 Gy curves, the position of the maximum of the curves is almost the same, for all densities and doses : $T_{\text{max}}\approx 35$ h (see figure 3); the duration of the repair phase is around 20 h and we find that $T_{\text{max}}-T_{a}$ is close to 15 h. Finally, based on this measure and on the profile likelihood of this parameter presented in figure 5*b*, which has a clear minimum, we fix γ=0.06h${,}^{-1}$ for all doses and initial cell densities.

We stress here that the simplification of the model by fixing γ to a common value for all doses and initial cell densities gives excellent results for the F98 cell line (as we show below) but may not be possible for other cell lines.

#### 3.3.2. Fitting data without irradiation

**Figure 6.**
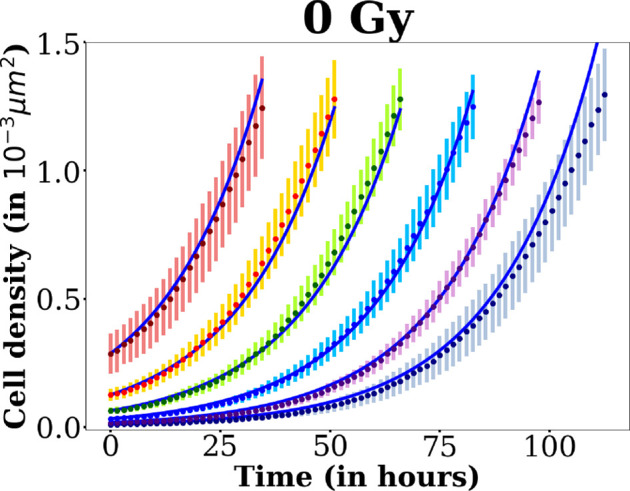
Cell densities versus time for 0 Gy for each seeding concentration (same as in figure 3). The fits for each seeding concentration are represented in blue. They are obtained with $k_{0}=0.045$ h${,}^{-1}$.

The best fit to the 0 Gy curves is a simple exponential one (see figure 6). To confirm this, we plot the natural logarithm of the normalized cell density at 0 Gy as a function of time (see electronic supplementary material, figure 4 and §5 ). We obtain a straight line, without any curvature, even for the curves corresponding to the highest cell densities (the red and yellow curves).

The exponential growth is a special case of logistic growth, when $C_{\text{max}}\gg C_{T}$. In this case the logistic term $1-C/C_{\text{max}}\approx 1$ for the range of cell densities considered here. Therefore, the precise value of $C_{\text{max}}$ cannot be deduced from fitting the 0 Gy curves.

The normal growth rate $k_{0}$ can be directly extracted from the experimental curves at 0 Gy, fitted with the function:


(3.12) $$\begin{array}{lcr} & C(t)=C_{0}e^{k_{0}t} & \end{array}$$

where $C_{0}$ represents the initial cell density. The averaged value obtained by the exponential fits on all of the seeding densities using large bounds is $k_{0}=0.045$ h${,}^{-1}$. We thus fix $k_{0}$ at this value for all the further fits, with different doses and initial cell densities. Fitting results are shown in figure 6.

We also check that the 0 Gy curves can be fitted to the model in response to radiation. To do so, the logistic term is removed and $T_{a}$ is set to 0. The fitting procedure leads to values of $\lambda_{u}$ between $10^{-3}$ and $10^{-13}$ depending on the initial cell density, with a profile likelihood showing minima approaching zero, and $k_{0}$ close to $k_{d}$, as expected (see electronic supplementary material, figures 11, 12 and §8). Hence, we can conclude that there is a continuity between the model with irradiation and the model without when the dose tends to zero.

#### 3.3.3. Fitting data with irradiation

If we now plot the natural logarithm of the normalized cell density at 5 Gy as a function of time (see electronic supplementary material, figure 4 and §5), a decrease of the slope (corresponding to a negative curvature) is clearly visible for the high density curves (the red and the orange ones). This means that to fit the curves at 5 Gy, a logistic term is necessary. This implies that the value of $C_{\text{max}}$ in the case of irradiation is much smaller than in the control case. This point is addressed in the discussion.

The fits on all seeding densities using large bounds for the 5 Gy curves yield an average value $C_{\text{max}}=2.4\;10^{-3}$ cells/µm${,}^{2}$, a value that is close to the maximum value reached by the cell densities at 5 Gy. For the 10 and 15 Gy curves, the low densities do not allow a reliable estimation of $C_{\text{max}}$, but longer experiments at 10 Gy reveal that at very long times (200 h), the cell density saturates at a maximum value similar to the one reached in the 5 Gy case. So we can reasonably fix $C_{\text{max}}$ to the 5 Gy value, for all the doses.

The model has seven parameters, and at this stage, four of them are fixed: $T_{a}$, γ, $C_{\text{max}}$ and $k_{0}$. We are left with only three free parameters, that have to be optimized with the fitting procedure, for all doses: $\left(\lambda_{s},\lambda_{u},\lambda_{r}\right)$, see table 1. We recall that we can calculate $k_{d}=\lambda_{u}+\lambda_{r}-\gamma$ and $\lambda_{D}=\gamma -\lambda_{s}$.

**Table 1.** List of model parameters.

	parameters	definition	fixed	bounds or value
control	*k*_0_	normal growth rate	yes	0.045 h^−1^
	γ	characteristic rate	yes	0.06 h${,}^{-1}$
	$C_{max}$	maximum cell density	yes	2.4 $10^{-3}$ cells/µm^2^
response to RT	$T_{a}$	duration of the repairing phase	yes	from 0 h (0 Gy) to 21 h (15 Gy)
	$\lambda_{u}$	passage rate from $C_{d}$ to $C_{u}$	no	[0;0.2] h${,}^{-1}$
	$\lambda_{r}$	passage rate from $C_{d}$ to $C_{r}$	no	[0;0.2] h${,}^{-1}$
	$\lambda_{s}$	Passage rate from $C_{u}$ to $C_{s}$	no	[0;0.06] h${,}^{-1}$

**Figure 7.**
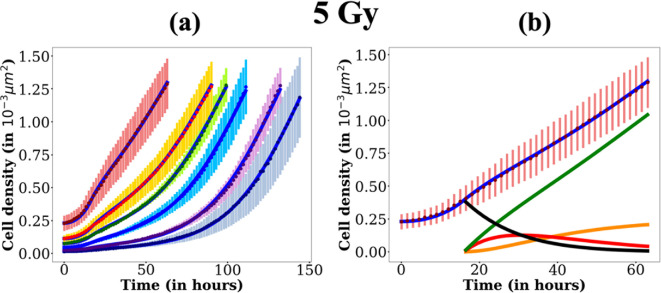
Cell densities versus time for 5 Gy (a) for each seeding concentration (same as for the control sample in figure 6). The corresponding fits are displayed in blue. In (b) is displayed the evolution of the cell density of each compartment ($C_{d}$ in black, $C_{u}$ in red, $C_{r}$ in green and $C_{s}$ in orange, as well as the overall cell density C in blue) on the experiment with the highest seeding concentration (3.52 $10^{5}$ cells/well). The fits are performed with $k_{0}=0.045$ h${,}^{-1}$, $C_{\text{max}}=2.4$ $10^{-3}$ cells/µm${,}^{2}$, $T_{a}=16$h, $\lambda_{s}\in \left[0;0.06\right]$ h${,}^{-1}$, $\lambda_{u}\in \left[0;0.2\right]$ h${,}^{-1}$ and $\lambda_{r}\in \left[0;0.2\right]$ h${,}^{-1}$ (see electronic supplementary material, §7 for the evolution of each compartment density for each seeding density)

**Figure 8.**
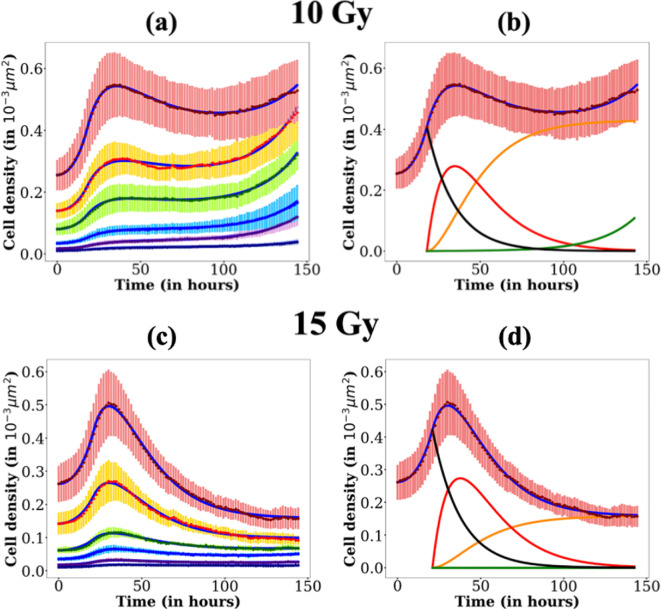
Cell densities versus time for 10 Gy (a) and (b) and 15 Gy (c) and (d), for each seeding concentration (same as in figure 3). The corresponding fits are displayed in blue. In (b) and (d) is displayed the evolution of the cell density of each compartment ($C_{d}$ in black, $C_{u}$ in red, $C_{r}$ in green and $C_{s}$ in orange, as well as the overall cell density C in blue) on the experiment with the highest seeding concentration (3.52 $10^{5}$ cells/well). The fits are performed with $k_{0}=0.045$ h${,}^{-1}$, $C_{\text{max}}=2.4$ $10^{-3}$ cells/µm${,}^{2}$, $T_{a}=18$h for 10 Gy and $T_{a}=21$h for 15 Gy, $\lambda_{s}\in \left[0;0.06\right]$ h${,}^{-1}$, $\lambda_{u}\in \left[0;0.2\right]$ h${,}^{-1}$ and $\lambda_{r}\in \left[0;0.2\right]$ h${,}^{-1}$ (see electronic supplementary material, §7 for the evolution of each compartment density for each seeding density).

Fixing γ implies constraining bounds for $\lambda_{s}$ since $\gamma =\lambda_{s}+\lambda_{D}$. Hence, the maximal value for $\lambda_{s}$ is set to 0.06 h${,}^{-1}$. The bounds used for each parameter are presented in table 1. Fitting results are presented in figure 7 for 5 Gy and in figure 8 for 10 and 15 Gy. An example of the evolution of each compartment determined by the model is also shown on the cell response curve with the highest seeding density. The values of the parameters are given in electronic supplementary material, section § of the Supplementary Material. We check that the fitting values of the parameters are indeed global minima by drawing, for each of them, their likelihood profile. The latter are presented for the 10 Gy case in electronic supplementary material, figure 11 §8.

### 3.4. Estimation of surviving fractions

Since clinicians typically rely on predictions of cell survival fractions after treatment—particularly through the LQ model—to inform their protocols, we sought to bridge our temporal model with this conventional approach. Specifically, we aimed to derive survival fraction estimates from our continuous model.

To obtain the surviving fractions, we have to estimate the fraction of the cells present at the beginning of the experiment ($C_{0}$) that are going to survive, i.e. end up ultimately in the $C_{r}$ compartment of well-repaired cells. To distinguish cells in $C_{r}$ coming from $C_{0}$ to those resulting from proliferation, we withdraw all types of proliferation in the model ($k_{d}=k_{0}=0$). This implies that nothing happens during the repairing phase meaning that at $t=T_{a}$, we have $C_{0}$ cells in $C_{d}$. Then, for $t\geq T_{a}$, as we do not have proliferation anymore but just a displacement of cells in the $C_{d}$ compartment to the $C_{r}$ and $C_{u}$ compartments, equation (3.2) becomes $dC_{d}/dt=-(\lambda_{u}+\lambda_{r})C_{d}.$ We can integrate this and implement the result in equation (3.3) to finally get the expression of the density of well-repaired cells as a function of time:


(3.13) $$\begin{array}{lcr} & C_{r}(t)=\frac{\lambda_{r}C_{0}}{\lambda_{u}+\lambda_{r}}(1-e^{-(\lambda_{u}+\lambda_{r})(t-T_{a})}) & \end{array}$$

This finally leads to ($t\geq T_{a})$:


(3.14) $$\begin{array}{lcr} & S(t)=\frac{\lambda_{r}}{\lambda_{u}+\lambda_{r}}(1-e^{-(\lambda_{u}+\lambda_{r})(t-T_{a})}) & \end{array}$$

where S corresponds to the surviving fraction.

Hence, when t→∞, $(t-T_{a})>>1/(\lambda_{u}+\lambda_{r})$, and we can estimate the surviving fraction by directly computing the ratio:


$$S=\frac{\lambda_{r}}{\lambda_{u}+\lambda_{r}}.$$

The resulting fractions are displayed in table 2. We show both the predicted fraction averaged over all the initial seeding densities and the predicted fraction averaged only over the two highest seeding densities (1.76 $10^{5}$ cells/well and 3.52 $10^{5}$ cells/wells).

**Table 2.** Predictions of the compartmental model for the surviving fraction of cells S in percentage for each radiation dose. The surviving fraction is averaged either on all initial cell densities (column ‘S - all $C_{0}$’) or the two highest seeding densities (column ‘S - 2 highest $C_{0}$’).

dose (Gy)	S - all $C_{0}$(in %)	S - 2 highest $C_{0}$(in %)
5	39±4	48±6
10	(6.5±1.2) $10^{-1}$	(4.2±1.4) $10^{-1}$
15	(1.8±1.0) $10^{-2}$	(4.4±3.0) $10^{-5}$

### 3.5. Effect of the cell density and the dose

On the experimental curves at 10 and 15 Gy (see figure 3), it seems that the population at the highest initial cell densities does not regrow as fast as for lower initial cell densities. To ensure that this effect is not due to the space constraint that would limit the regrowth in the wells with the highest cell density, we plot $C_{r}(t)$ and $C_{s}(t)$ obtained with the model (where the limitation due to the lack of space at high density is taken into account) normalized by their corresponding $C_{0}$, as a function of time, in the 10 Gy case (see electronic supplementary material, figure 13, §9). We notice that if values of $C_{s}/C_{0}$ stay close over the range of $C_{0}$ considered, the values of $C_{r}/C_{0}$ stretch on a large range.

**Figure 9.**
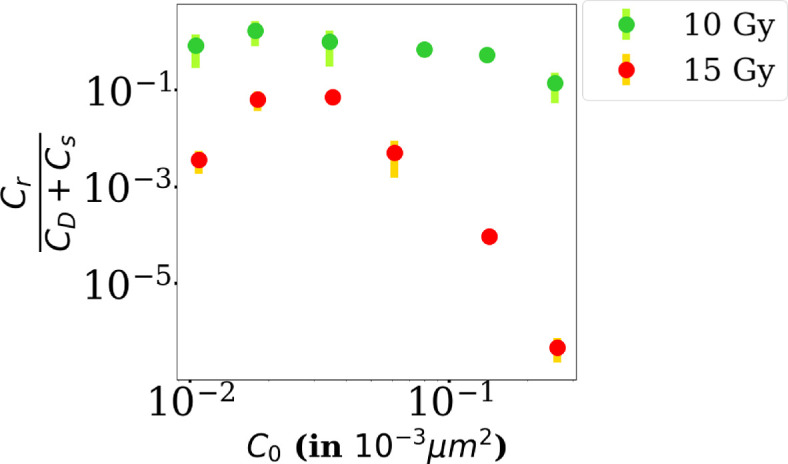
Ratio of well-repaired cells $C_{r}$ to the total amount of unrepaired cells $\left(C_{s}+C_{D}\right)$ predicted by the model at the endpoint of the experiment (*t* =144 h) as a function of the initial cell density $C_{0}$ for 10 and 15 Gy. Each data point represents the average value obtained by the fit over three independent experiments.

From these results, it is clear that the ratio $C_{r}/C_{s}$ depends on the initial cell density. To study whether the unrepaired population ($C_{s}$ and $C_{D}$) has an influence on the well repaired $C_{r}$ population, we computed the ratio of $C_{r}$ to the total amount of unrepaired cells $\left(C_{s}+C_{D}\right)$ as a function of the seeding cell density $C_{0}$, at 6 days, as predicted by the model (see figure 9), for 10 and 15 Gy (the densities reached at 6 days with the low dose of 5 Gy being higher than the threshold $C_{T}$). As expected, the ratio decreases with the dose for every seeding density. But, more interestingly, we observe that the ratio tends to decrease at higher densities and that this effect is particularly emphasized at high dose (15 Gy).

**Figure 10.**
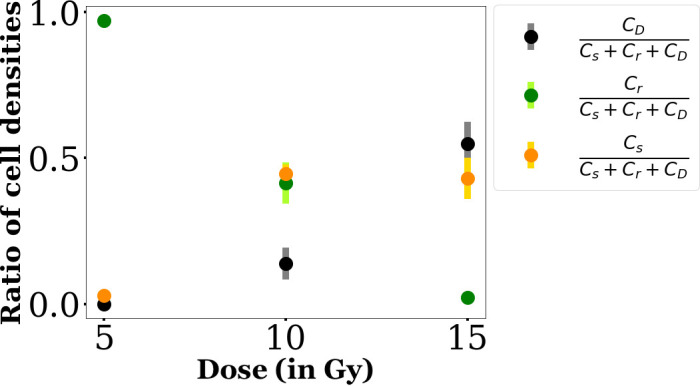
Ratios of the number of cells in the different final compartments, $C_{r}$, $C_{s}$ and $C_{D}$, to the total amount of cells in these three predicted by the model at the endpoint of the experiment (*t* =144 h) as a function of the dose. The values are averaged over the ones obtained for each seeding densities, with the exception of the 5 Gy case where we can only have access to the value of the lowest density at 144 h.

To investigate further the effects of RT, we also plotted the ratios $C_{r}/(C_{s}+C_{D}+C_{r})$, $C_{s}/(C_{s}+C_{D}+C_{r})$ and $C_{D}/(C_{s}+C_{D}+C_{r})$ at 6 days as a function of the dose. We show the values obtained for the lowest initial density of 5 Gy and the averaged values obtained from the values for all initial densities for 10 and 15 Gy in figure 10. This kind of plot brings out the effect of the dose: the higher the dose, the lower the amount of well-repaired cells and the higher the amount of dead cells. For senescent cells, it seems that below 10 Gy, the amount of senescent cells increases with the dose whereas beyond 10 Gy, the cells rather die than become senescent (however, we lack data points to be able to clearly conclude).

## 4. Discussion

We developed a mathematical model based on cell population compartments characterizing the time-dependent response of F98 glioma cells to a single dose of X-rays. Time-lapse fluorescence microscopy was used to quantify this evolution over a broad range of seeding densities and radiation doses.

One of the main strengths of our experimental approach is the fact that we directly count cells instead of estimating their confluence. This gives data that are closer to the real evolution of the cell population. Indeed, in response to irradiation, particularly at high doses (above 10 Gy), the surface area of the nuclei becomes very inhomogeneous. The confluence can actually increase whereas the number of cells does not. Our approach has the advantage of avoiding this flaw. Yet, one drawback is the fact that the intensity of the nuclei can become very heterogeneous at large time and high doses which leads to the detection of several maxima per nucleus instead of one. This is why we did not include the data at 20 Gy in the present study.

Regarding our theoretical approach, we were able to fit all the data corresponding to different doses of radiation and different initial cell densities with excellent agreement, with only three free parameters. We believe this model to be biologically meaningful, particularly in its ability to incorporate the temporal dynamics of crucial radiation-induced mechanisms like cell-cycle arrest and mitotic catastrophe [39–41,41–44,46]. Notably, we find that the duration of the repairing phase $T_{a}$ (which is the phase where the DSBs are repaired) is close to 20 h, which is in good agreement with the 24 h value often reported in the literature for experiments quantifying the protein γH2AX [28,40,50].

Compared to other modelling approaches, such as in [28], the compartmental model allows us to track the dynamic evolution of the different cell states over time (damaged, unrepaired, senescent and repaired cells), which can help to understand the role of each cell population in the regrowth of the whole population. In particular, we show that the ratio of repaired to unrepaired cells (as predicted by the model) decreases when the initial cell density increases, for all doses between 5 and 15 Gy, six days after irradiation (see figure 3). This result is consistent with the conclusion of [20], where the authors show that the surviving fraction of breast cancer cells after a high single dose (12 Gy) depends on the number of cells irradiated per flask. Dying or senescent cells could inhibit (or delay) the proliferation of repaired cells, via a transferable factor in the medium or intercellular communication. This is a cohort effect (i.e. an effect on irradiated cells caused by other irradiated cells within the target volume [51,52]). To our knowledge, it is the first time that a model is used to infer the different cell populations after irradiation (instead of costly and time-consuming immunostaining) and reveals a cohort effect. The cohort effect we highlight here would increase the efficacy of irradiation at high doses, which would be an argument to develop research on the high-dose modalities in radiotherapy.

Using the dynamic of the different cell states in our model, we were also able to estimate surviving fractions at large time after irradiation, which are of primary interest for clinicians. Even if the simulated surviving fractions are effective ones (for example, including a delay of regrowth after $T_{a}$ for the $C_{r}$ cells would modify the values of the surviving fractions), the important point is that they also depend (as the repaired-unrepaired ratio) on the initial cell density. This effect cannot be taken into account in clonogenic assays used to determine the parameters of the LQ model. Due to the fact that the simulated surviving fractions could be different with a different model, a more precise quantitative comparison between the simulated surviving fractions and the surviving fractions predicted by the LQ model would not be insightful.

The fact that the surviving fractions and their time course seem to depend on the initial cell density could have important clinical consequences, because *in vivo*, in gliomas, the tumour cell density is very spatially heterogeneous: it is low at the border of the tumour (in the invasion area) and it increases towards the centre. Since new modalities of radiotherapy use high doses (≥10 Gy), it is of the utmost importance to understand how the cell density can modulate the effect of radiotherapy, in particular at high dose.

Another interesting outcome of our experiments is the fact that in the range of densities considered here ($C\leq 1.3\;10^{-3}$ cells/µm${,}^{2}$, or densities smaller than 50 % of the confluence), non irradiated cells seem to grow without being hindered by the presence of other cells, whereas irradiated cells display a reduced growth due to the presence of other cells very early. This translates in the model to the fact that the growth of the non irradiated cells is exponential, whereas a logistic growth best describes the growth of the irradiated population. A possibility is that the non irradiated cells climb on each other, forming three-dimensional clusters. Irradiated cells have an altered cytoskeleton and their adhesion to the substrate is stronger [53]; they are also much bigger than their non-irradiated counterparts. It is thus plausible that as a consequence, they lose their ability to form three-dimensional structures. If they can grow only in two dimensions, their growth is rapidly hindered by the presence of neighbouring cells. We will test this hypothesis in a future work.

Our results will have to be generalized to other cell lines. Since the qualitative shape of the temporal evolution of cell density for F98 cells is similar to those reported for different glioma cell lines [28], we are confident that our model will be able to reproduce the experimental evolution of these other glioma cell populations. Still, it would be interesting to confront it to cell lines from other cancers and even normal cell lines. The negative influence of the senescent/dying cells on the growth of the repaired population will also have to be explored with other cell lines (such an effect has not been reported by [28]). We are also planning to study more realistic three-dimensional experimental models, such as spheroids, and we believe that our model of the cell response to irradiation would be easily incorporated into a more complex biological model of tumour response to RT.

## Data Availability

Data and relevant code for this research work are stored in Dryad [54]. Supplementary material is available online [55].
